# Potential Role of the Mitochondria for the Dermatological Treatment of Papillon-Lefèvre

**DOI:** 10.3390/antiox10010095

**Published:** 2021-01-12

**Authors:** Beatriz Castejón-Vega, Maurizio Battino, José L. Quiles, Beatriz Bullon, Mario D. Cordero, Pedro Bullón

**Affiliations:** 1 Research Laboratory, Dental School, University of Sevilla, 41009 Sevilla, Spain; beacastej92@outlook.es; 2 Dipartimento di Scienze Cliniche Specialistiche ed Odontostomatologiche, Sez. Biochimica, Università Politecnica delle Marche, 60131 Ancona, Italy; m.a.battino@staff.univpm.it; 3 International Research Center for Food Nutrition and Safety, Jiangsu University, Zhenjiang 212013, China; 4 Department of Physiology, Institute of Nutrition and Food Technology “José Mataix”, Biomedical Research Center (CIBM), University of Granada, Avda. del Conocimiento s.n., 18100 Armilla, Spain; jlquiles@ugr.es; 5 Research Group on Food, Nutritional Biochemistry and Health, Universidad Europea del Atlántico, 39011 Santander, Spain; 6 Department of Periodontology, Dental School, University of Sevilla, 41009 Sevilla, Spain; beatrizbullon@hotmail.com; 7 Instituto de Investigación e Innovación en Ciencias Biomédicas de Cádiz, INiBICA, 11009 Cadiz, Spain; 8 Cátedra de Reproducción y Genética Humana del Instituto para el Estudio de la Biología de la Reproducción Humana (INEBIR)-Universidad Europea del Atlántico (UNEATLANTICO)-Fundación Universitaria Iberoamericana (FUNIBER), 39011 Santander, Spain

**Keywords:** Papillon–Lefèvre syndrome 1, mitochondria 2, coenzyme Q10 3

## Abstract

The Papillon–Lefèvre syndrome (PLS) is a rare autosomal recessive disorder caused by mutations in the Cathepsin C (CTSC) gene, characterized by periodontitis and palmoplantar hyperkeratosis. The main inflammatory deficiencies include oxidative stress and autophagic dysfunction. Mitochondria are the main source of reactive oxygen species; their impaired function is related to skin diseases and periodontitis. The mitochondrial function has been evaluated in PLS and mitochondria have been targeted as a possible treatment for PLS. We show for the first time an important mitochondrial dysfunction associated with increased oxidative damage of mtDNA, reduced CoQ10 and mitochondrial mass and aberrant morphologies of the mitochondria in PLS patients. Mitochondrial dysfunction, determined by oxygen consumption rate (OCR) in PLS fibroblasts, was treated with CoQ10 supplementation, which determined an improvement in OCR and a remission of skin damage in a patient receiving a topical administration of a cream enriched with CoQ10 0.1%. We provide the first evidence of the role of mitochondrial dysfunction and CoQ10 deficiency in the pathophysiology of PLS and a future therapeutic option for PLS.

## 1. Introduction

Papillon–Lefèvre syndrome (PLS) is a rare autosomal recessive condition characterized by severe early-onset periodontitis, leading to premature loss of primary or secondary teeth, and palmoplantar hyperkeratosis [1]. Dermatological symptoms can begin prior to 2 years of age and continue throughout life and are sometimes accompanied by an increased susceptibility to infections [2]. The main problem is to identify the cellular pathogenic mechanisms that could explain the disease. More than 30 different mutations of the Cathepsin C (CTSC) gene have been described in patients with PLS that result in a complete loss of CTSC activity [3]. CTSC is an oligomeric lysosomal cysteine protease that removes the inhibitory N-terminal dipeptide. An important step in cellular physiology is the synthesis of different molecules, such as proteins, but also the removal of the inhibitory N-terminal dipeptide is essential. The cellular turnover of proteins, organelles and microorganisms require the cooperation between both autophagy machinery and lysosomal degradation [4]. Fibroblasts in PLS patients show an autophagic dysfunction that is associated with autophagosome alterations and lysosomal permeabilization and is restored with recombinant CTSC treatment [5]. Other cellular disorders have been reported, including oxidative stress with an impairment in the antioxidant capacity of the subjects characterized by abnormally high hydroperoxide levels and altered CoQ and vitamin E contents [6]. Furthermore, the antimicrobial activity of neutrophils which is mediated by the neutrophil extracellular trap formation and the production of reactive oxygen species is abrogated in PLS [7]. Mitochondria are the main source of reactive oxygen species that induce oxidative stress which is involved in the short-term regulation of mitochondrial morphology and function [8]. Moreover, the main feature in PLS is that the periodontitis is related to multifactor phenomena such as free radicals, reactive oxygen species and mitochondrial dysfunction [9]. Emerging evidence suggests that mitochondria are vital regulators of skin physiology, and mitochondrial dysfunction and aberrant structure of mitochondria are associated to skin alterations in different rare diseases with mitochondrial mutations. Regarding this, therapeutic targeting of mitochondria must be considered in the treatment of a wide range of skin diseases [10]. Our aim is to study the mitochondria in PLS that have never been studied and we evaluate the mitochondrial function in PLS and target mitochondria as a possible treatment for PLS.

## 2. Materials and Methods

### 2.1. Ethical Statements

Approval of the ethical committee of the University of Seville was obtained, according to the principles of the Declaration of Helsinki and to all the International Conferences on Harmonization and Good Clinical Practice Guidelines (13/03/12). All the participants in the study gave their written informed consent before initiating it.

### 2.2. Reagents

Anti-GAPDH monoclonal antibody from Calbiochem-Merck Chemicals Ltd. (Nottingham, UK). Anti-OGG1 from Santa Cruz Biotechnology. Trypsin was purchased from Sigma Chemical Co. (St. Louis, MO, USA). A cocktail of protease inhibitors (complete cocktail) was purchased from Boehringer Mannheim (Indianapolis, IN, USA). The Immun Star HRP substrate kit was from Bio-Rad Laboratories Inc. (Hercules, CA, USA).

### 2.3. Fibroblast Cultures

Patients and control fibroblasts were obtained by protocols according to the Helsinki Declarations revised in 2001. Two control fibroblast lines were used. Fibroblasts were cultured in high glucose DMEM (Dulbecco’s modified media) (Gibco, Invitrogen, Eugene, OR, USA) with 10% of fetal bovine serum (FBS) (Gibco, Invitrogen, Eugene, OR, USA) and 5% of antibiotics (Sigma Chemical Co., St. Louis, MO, USA) FIbroblasts were incubated at 37 °C in a 5% CO_2_ atmosphere.

### 2.4. Antioxidant Enzyme Activity

Enzymatic activity of catalase and SOD were determined in 1 × 10^6^ cells. Catalase activity was determined from cellular lysate by monitoring H_2_O_2_ decomposition at 240 nm 13. SOD activity was determined from the basis of the inhibition of the formation of NADH−phenazine methosulfate-nitroblue tetrazolium formazan.

### 2.5. Measurement of CoQ10 Levels

Methodology of the lipid extraction from fibroblasts was performed as described previously [6]. Briefly, cells were lysed with 1% SDS and vortexed for 1 min. A combination of ethanol:isopropanol (95:5) was added and were vortexed for 1 min. Five ml of hexane was added and samples were centrifuged at 1000× *g* for 5 min at 4 °C to recover CoQ10. The upper phases from three different extractions were recovered and dried by a rotatory evaporator (Rotavapor^®^ R-210, BÜCHI Labortechnik AG, Flawil, Switzerland). Lipid extract was resuspended in 1 mL ethanol, dried in a speed-vac, and kept at −20 °C until used. Samples were suspended in 60 µL of ethanol to be injected in HPLC.

### 2.6. Western Blotting

Whole cellular lysate from cells was prepared by gentle shaking with a lysis buffer containing 0.9% NaCl, 0.1% triton X−100, 20 mM Tris-ClH, pH 7.6, 1 mM phenylmethylsulfonylfluoride and 0.01% leupeptine. Electrophoresis was carried out in a 10–15% acrylamide SDS/PAGE and proteins were transferred to Immobilon membranes (Amersham Pharmacia, Piscataway, NJ, USA). After this, membranes were washed with PBS, blocked over night at 4 °C and incubated with primary antibodies at 1:1000. Then, membranes were incubated with secondary antibody (1:2500). Immunolabeled proteins were detected by chemiluminescence method (Immun Star HRP substrate kit, Bio-Rad Laboratories Inc., Hercules, CA, USA) and quantified using ImageJ software (see: http://rsb.info.nih.gov/ij/download.html).

### 2.7. Measurement of Citrate Synthase Activity

Citrate synthase specific activity in fibroblast extracts was measured at 412 nm minus 360 nm (extinction coefficient 13.6 mM^−1^ cm^−1^) using 5,5-dithio-bis (2-nitrobenzoic acid) to detect free sulfhydryl groups in coenzyme A as previously was described [6].

### 2.8. Mitochondrial ROS Production

Mitochondrial ROS generation in fibroblasts was assessed by MitoSOX™ red according to the protocol my microscopy and flow cytometry.

### 2.9. Oxygen Consumption Rate (OCR)

Oxygen consumption rate (OCR) was assessed in real-time using the Extracellular Flux Analyzer XF-24 (Seahorse Bioscience, North Billerica, MA, USA) according to the manufacturer’s protocol, and previously described [5].

### 2.10. Electron Microscopy

Fibroblasts were fixed with 1.5% glutaraldehyde for 15 min in the culture plates with culture medium and then for 30 min in 1.5% glutaraldehyde-0.1 M NaCacodylate/HCl, pH 7.4. They were then washed three times in 0.1 M NaCacodylate/HCl, pH 7.4 during 10 min each. Then. Cells were post-fixed with 1% OsO_4_-H_2_O, pH 7.4 for 30 min. After dehydration in increasing concentrations of ethanol, 50, 70, 90 and three times 100%, impregnation and inclusion steps were performed in Epon and polymerized at 60 °C for 48 h. After obtaining 60–80 nm sections by ultramicrotome Leica ultracut S (Leitz Microsystems, Wetzlar, Germany) and contrasted with uranyl acetate and lead citrate, samples were observed on a Zeiss LEO 906 E (Oberkochen, Germany) transmission electron microscope.

### 2.11. Proliferation Rate

Two hundred thousand fibroblasts from control and patients were cultured in the absence or presence of CoQ10 for 24, 48, 72 and 96 h. After discharging supernatant, cell counting was performed from 3 high power fields using an inverted microscope and a 40× objective and quantified.

### 2.12. PCR Amplification and Sequencing

mtDNA was amplified from total DNA in 24 overlapping 800–1000 bp-long PCR fragments. Primers were designed using the revised human mtDNA Cambridge Reference Sequence (http://www.mitomap.org/mitoseq.html). PCR fragments were sequenced in an ABI 3730 (Applied Biosystems; http://www.appliedbiosystems.com; Foster City, CA, USA) sequencer using the BigDye V.3.1 Sequencing Kit (Applied Biosystems; http://www.appliedbiosystems.com). Assembling and identification of the variations in the mtDNA were carried out using the Staden package [11]. For this purpose, the revised human mtDNA Cambridge Reference Sequence (http://www.mitomap.org/mitoseq.html) was used. The whole process was carried out at Secugen (Madrid, Spain).

### 2.13. Statistical Analysis

Data in the figures is given as mean ± SD. Data between different groups were analyzed statistically by using ANOVA on Ranks with Sigma Plot and Sigma Stat statistical software (SPSS for Windows, 19, 2010, SPSS Inc., Chicago, IL, USA). For cell-culture studies, Student’s *t* test was used for data analyses. A value of *p* < 0.05 was considered significant.

## 3. Results

Patient 1 was a 21-year-old woman with hyperkeratosis in the palmoplantar region and joints and moderate periodontitis. Genetic characterization showed that the patient was homozygote for a nonsense CTSC mutation, (Table S1). This genetic change was observed in the mother and father in heterozigosis without phenotypic manifestation, without genetic changes in the brother (data not shown). Patient 2 was a 31-year-old man with hyperkeratosis in the palmoplantar region of his hands and moderate periodontitis. Genetic characterization revealed that the patient was a compound heterozygote for two nonsense CTSC mutations (Table S1). Both genetic changes were observed in the mother and father respectively without phenotypic manifestation, without genetic changes in the brother (data not shown).

Analysis of the entire mitochondrial genome revealed several polymorphic variants in both patients compared with control and with the revised Cambridge Reference Sequence (Figures S1 and S3). Interestingly, patients showed a high similarity ratio with the sequence of the mothers showing a typical maternal inheritance (Figures S2 and S4).

**Figure 1:**
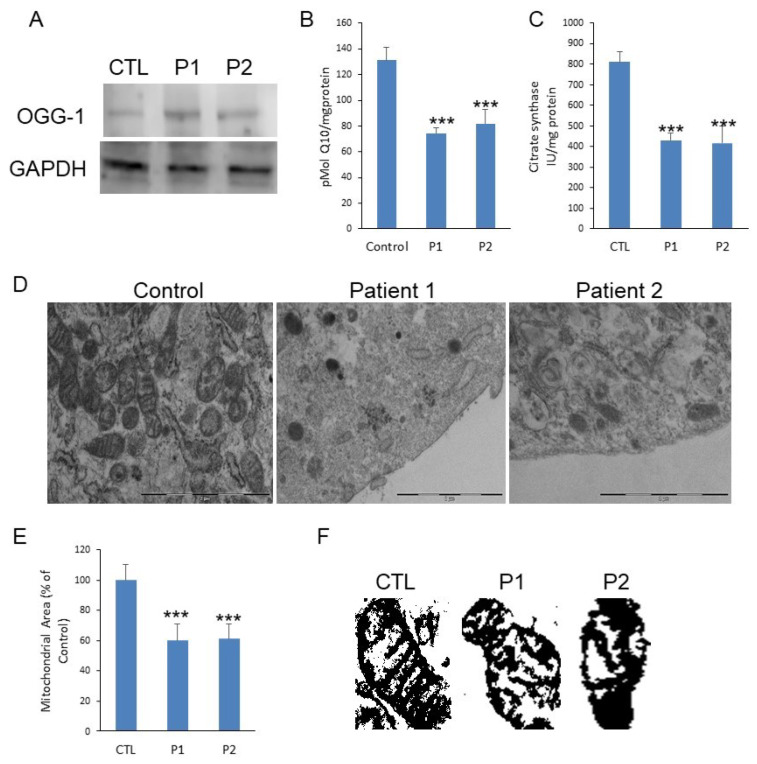
(**A**). Protein expression levels of OGG-1 were determined in Papillon-Lefevre Syndrome fibroblast cultures by Western blot analysis compared with a pool of two control subjects, as described in Materials and Methods. GAPDH was used as a loading control. (**B**). CoQ10 levels were measured by HPLC. Data represent the mean ± SD of three separate experiments. (**C**). Citrate Synthase specific activity in fibroblasts from PLS fibroblast compared with a pool of two healthy age-matched and sex-matched control subjects, was performed by enzymatic activity. Data represent the mean ± SD of three separate experiments. * *p* < 0.05; ** *p* < 0.01; *** *p* < 0.001 between control and PLS patients (**D**). Transmission electron microscopy of representative control fibroblasts showing mitochondria with typical ultrastructure and number and low number of mitochondria present in PLS fibroblasts with aberrant structure. Scale bar 2 µm. (**E**). Mitochondrial area of PLS patients compared with control fibroblasts. (**F**). Representative masks of individual fibroblasts mitochondria from control and patients to show aberrant structure with alterations of cristae.

The expression levels of 8-oxoguanine glycosylase (OGG-1), a DNA glycosylase enzyme responsible for the excision of 7,8-dihydro-8-oxoguanine (8-oxoG), a mutagenic base byproduct result of exposure to ROS. The patients showed increased levels of OGG-1 (Figure 1A). The patients also showed reduced levels of Coenzyme Q10 (CoQ10) (Figure 1B). Patients showed reduced citrate synthase, which propose a reduced mitochondrial mass in PLS (Figure 1C). Ultrastructure analysis by transmission electron microscopy (TEM) confirmed the reduced number of mitochondria (Figure 1D) and shows morphological modifications such as cristae alterations (Figure 1D–F).

**Figure 2:**
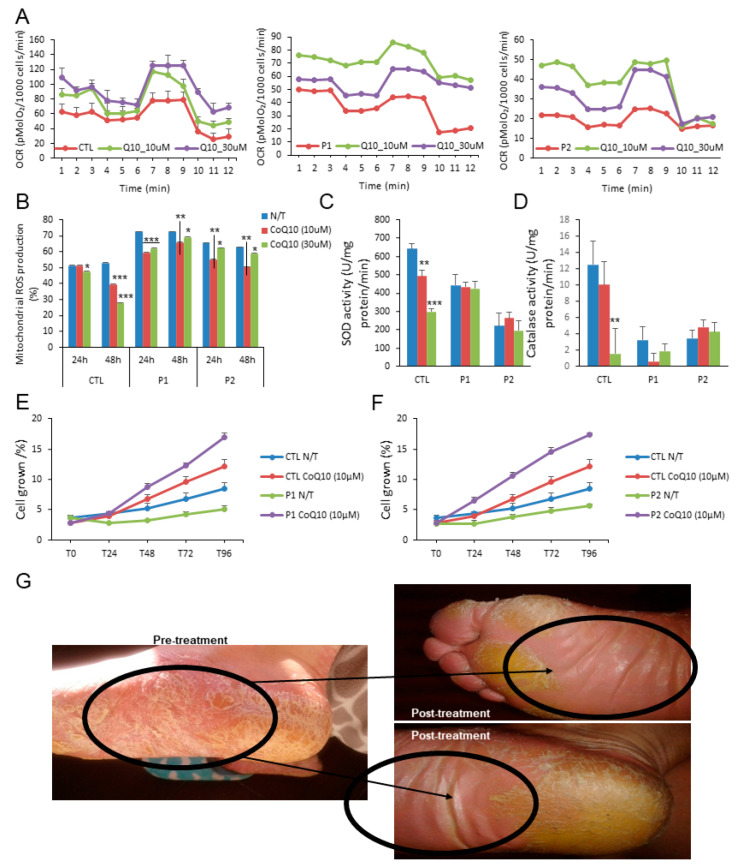
Effect of CoQ10. (**A**). Oxygen consumption rate (OCR) in cells from control (mean of two controls) and PLS patients with and without CoQ10. OCR was monitored using the Seahorse XF-24 Extracellular Flux Analyzer with the sequential injection of oligomycin (2.5 μg/mL), 2,4-DNP (1 mM), antimycin (10 μM)/rotenone (1 μM) at the indicated time point. (**B**). Effect of CoQ10 treatment in mitochondrial ROS production of patients and control (% mean percentage of ROS production with respect to control). (**C**,**D**). Antioxidant enzymes SOD and catalase (CAT) activities were also analyzed in fibroblasts from control and PLS patients treated with and without CoQ10. (**E**) (patient 1) and (**F**) (patient 2). The effect of CoQ10 in cell growth determined in healthy and PLS fibroblasts. % is the percentage of the cellular growth per day at 4 days with respect to first day. For control cells, the data are the mean ± SD of the two control subjects cell lines. Data represent the mean ± SD of three separate experiments. * *p* < 0.05; ** *p* < 0.01; *** *p* < 0.001 between CoQ10 treatment and no treatment. (**G**). Representative image of the effect of CoQ10 in the plantar region of a patient after 60 days.

The measurements of the protective effect of CoQ10 on mitochondrial functionality of PLS by measuring the OCR values in control and treated cells show that after 24 h of treatment the mitochondrial dysfunction determined by OCR was restored by two different doses of CoQ10 (10 µM and 30 µM) (Figure 2A). This metabolic improvement was accompanied by a decrement of mitochondrial ROS production by both doses in the patients after 24 and 48 h (Figure 2B); however, no changes in the reduced activity of the antioxidant enzymes, superoxide dismutase and catalase were observed (Figure 2C,D). Despite this, the decrease in cell proliferation was enhanced by CoQ10 (Figure 2E,F). As a proof of concept, the patients were treated with a topical cream enriched with CoQ10 0.1%. Importantly, after 60 days of treatment, a remission of skin damage was observed (Figure 2G).

## 4. Discussion

In the past years, most studies about PLS focused on the description of new CatC mutations. Recently, several descriptions of the pathophysiological alterations associated with PLS such as oxidative stress and neutrophil function impairment have been reported. The mitochondrial function is an essential mechanism for the survival of cells under different conditions. In this report, we show for first time an important mitochondrial dysfunction associated with increased oxidative damage of mtDNA, reduced CoQ10 and mitochondrial mass and aberrant morphologies of the mitochondria in PLS patients. All these alterations could explain the metabolic dysfunction and low bioenergetics previously shown by our group [5]. Furthermore, a mitochondrial dysfunction could be associated with the impaired neutrophil function shown in PLS [12,13]. Our data report mitochondrial damage in PLS patients with reduced mitochondrial mass and histological degeneration, as a dysfunctional marker, related to oxidative stress determined by high levels of OGG1 and reduced CoQ10. OGG1 is a mutagenic base byproduct resulting from exposure to ROS and indicative of oxidative stress. CoQ10 plays a crucial role in cellular metabolism acting as an electron carrier between complexes I and II and complex III of the mitochondrial respiratory chain, and is suggested to be useful as a mitochondrial dysfunction marker [14]. These reported data are the expression of mutations in mtDNA related to a wide range of human pathologies which show skin pathophysiology [10]. mtDNA is more susceptible than nuclear DNA to oxidative damage and is the cause of the high level of heteroplasmy [15]. The mitochondrial damage induces oxidation of mtDNA that can be released to the cytosol and can lead to inflammasome activation, IL-1β secretion and synthesis of mtDNA which modulate NLRP3 signalling [16]. It has been shown in an experimental arthritis induced model that mtDNA displays inflammatogenic properties as a result of its oxidatively damaged adducts [17]. Furthermore, it has been demonstrated that autophagy deficiencies overstate mitochondrial injuries [18]. So, mitochondria are an essential part of the inflammatory reaction in PLS.

Due to the lack of effective therapeutic tools for PLS, the results presented in this report propose the therapeutic effect of CoQ10, on fibroblasts and in the clinical improvement. In the in vitro experiments we used two different concentrations to be sure of the mitochondrial effects. The reason for it that usually, the CoQ10 concentration is 10 µmol [19], but it has been known that low doses cannot produce metabolic effects [20] so we used 30 µmol. CoQ10 produces a reduction in ROS but not in SOD and catalase values SOD and catalase are other antioxidants enzymes, so it is logical to suppose that CoQ10 cannot modulate them, but it can change the ROS level indicating an improvement in mitochondrial physiology. CoQ10 supplementation has been shown to improve the pathophysiology in aging [21], cardiovascular and metabolic diseases [22], and in psoriasis, a dermatological disease with alterations in the epithelial maturation [23]. Therefore, treatment of PLS with CoQ10 could be appropriate. However, we are aware of the limitation of our study. The most important is the reduced number of patients. However, we have to consider that is very rare disease and we used techniques that require the collaboration of the patients to take biological samples. We recognize that is a preliminary study with a reduced number of patients that these data need to be supported with new studies, but there can used it to design new protocols in the psoriatic diseases.

## 5. Conclusions

Mitochondrial homeostasis depends mainly on the statement of the electron transport chain and the oxidation metabolic process. CoQ10 is one of the main antioxidants molecule and its level has been related to mitochondrial disfunction. Therefore, our mitochondrial dysfunction results and the restoration with CoQ10 can be proposed as a therapeutic option for this type of patients. We provide the first evidence of the role of mitochondrial dysfunction and CoQ10 deficiency in the pathophysiology of PLS and provide a new insight into the pathogenesis and a future therapeutic option for PLS and similar dermatological diseases. A randomized, controlled trial comparing CoQ10 treatment with placebo and with other conventional agents to verify the efficacy demonstrated in this initial report is urgently needed.

## Data Availability

Data is contained within the article or Supplementary Material.

## Supplementary Materials

The following are available online at https://www.mdpi.com/2076-3921/10/1/95/s1.
