# Supplementary material for: Potential Role of the Mitochondria for the Dermatological Treatment of Papillon-Lefèvre
Source: Antioxidants (Basel). 2021 Jan 12;10(1):95. doi: 10.3390/antiox10010095 (PMC7827181; doi:10.3390/antiox10010095)
Supplement: Supplementary file 1 [file antioxidants-10-00095-s001.pdf]

antioxidants

Supplementary Materials:

Potential role of the mitochondria for the dermatological treatment of Papillon Lefevre

Table S1. Characteristic findings of PLS patients and controls

	Control 1	Control 2	Patient 1	Patient 2
Age (yr)	42	41	21	21
Periodontal damage	No	No	Yes	Yes
Dermatological damage	No	No	Yes	Yes
Sex	Male	Male	Female	Male
CTSC Mutation	No	No	c.1286>A	c.96T>G and c.401G>A
Amino acid change	No	No	p.W429*	p.Y32* and p.W134*

Table S2. Summary of sequencing results in control fibroblasts.

	**Control 1**	**Control2**
MT-RNR1	A750G A1438G	A750G A1438G
MT-RNR2	A2706G T3197C	
MT-ND1	A3564G	T3777C G3915A
MT-ND2	A4769G	A4727G A4769G
MT-CO1	A6040G C7028T	
MT-CO2		G7805A
MT-TS1		

MT-ATP6	T8610C<br>G8838A<br>A8860G	A8860G
MT-CO3		G9380A
MT-ND4	C11054T<br>A11467G<br>G11719A	T11253C
MT-ND5	G12376A<br>T13617C	
MT-CYB	C14766T<br>A15326G	A15326G
MT-TT		
MT-DLOOP	C16192T<br>C16256T<br>C16270T<br>A16399G<br>T16519C<br>A73G<br>A263G<br>310insC	T16362C<br>A16482G<br>C150T<br>T239C<br>A263G<br>302insCC<br>310insC<br>514inCA

Figure S1: mtDNA sequence from Patient 1

MT-RNR1	m.750A>G
	m.1438A>G
MT-RNR2	m.2706A>G
MT-ND1	m.4216T>C (Tyr304His)
MT-ND2	m.4769A>G (Met100Met)
	m.5484A>G (Ile339Val) (heteroplasmia)
	m.5493T>C (Phe342Leu) (heteroplasmia)
MT-CO1	m.6671T>C (His256His)
	m.7028C>T (Ala375Ala)
	m.7076A>G (Gly391Gly)
MT-CO2	
MT-TS1	m7476C>T
MT-ATP6	m.8860A>G (Thr112Ala)
	m.8958C>T (Ile144Ile)
MT-CO3	
MT-ND3	m.10398A>G (Thr114Ala)
MT-ND4L	m.10499A>G (Leu10Leu)
MT-ND4	m.11002A>G (Gln81Gln)
	m.11016G>A (Ser86Asn)
	m.11251A>G (Leu164Leu)
	m.11377G>A (Lys206Lys)
	m.11719G>A (Gly320Gly)
MT-TL2	

Figure S2. mtDNA sequence from family of patient 1.

FATHER

Mutación	120930/JRS
MT-RNR1	m.750A>G
	m.1438A>G
MT-RNR2	m.1811A>G
	m.2706A>G
MT-ND1	m.3720A>G (Gln138Gln)
MT-ND2	m.4769A>G (Met100Met)
	m.5390AG (Met307Met)
	m.5426T>C (His319His)
MT-CO1	m.6045C>T (Leu48Leu)
	m.6152T>C (Val83Val)
	m.7028C>T (Ala375Ala)
MT-CO2	
MT-TS1	
MT-ATP6	m.8860A>G (Thr112Ala)
MT-CO3	
MT-ND3	
MT-ND4L	
MT-ND4	m.10876A>G (Leu39Leu)
	m.11467A>G (Leu236Leu)
	m.11719G>A (Gly320Gly)
MT-TL2	m.12308A>G
MT-ND5	m.12372G>A (Leu12Leu)
	m.13020T>C (Gly228Gly)
	m.13734T>C (Phe466Phe)
MT-CYB	m.14766C>T (Ile7Thr)
	m.15326A>G (Thr194Leu)
MT-TT	m.15907A>G
MT-DLOOP	m.16051A>G
	m.16129G>C
	m.16183A>C
	m.16189T>C
	m.16362T>C
	m.16519T>C
	m.73A>G
	m.152T>C
	m.217T>C
	m.263A>G
	m.302insCC
	m.310insC
	m.340C>T
	m.508A>G
	m.514insCA

MOTHER

Mutación	120929/MPV
MT-RNR1	m.750A>G
	m.1438A>G
MT-RNR2	m.2706A>G
MT-ND1	m.4216T>C (Tyr304His)
MT-ND2	m.4769A>G (Met100Met)
	m.5484A>G (Ile339Val)(heteroplasmia)
	m.5493T>C (Phe342Leu) (heteroplasmia)
MT-CO1	m.6671T>C (His256His)
	m.7028C>T (Ala375Ala)
	m.7076A>G (Gly391Gly)
MT-CO2	
MT-TS1	m7476C>T
MT-ATP6	m.8860A>G (Thr112Ala)
	m.8958C>T (Ile144Ile)
MT-CO3	
MT-ND3	m.10398A>G (Thr114Ala)
MT-ND4L	m.10499A>G (Leu10Leu)
MT-ND4	m.11002A>G (Gln81Gln)
	m.11016G>A (Ser86Asn)
	m.11251A>G (Leu164Leu)
	m.11377G>A (Lys206Lys)
	m.11719G>A (Gly320Gly)
MT-TL2	
MT-ND5	m.12570A>G (Leu78Leu)
	m.12612A>G (Val92Val)
	m.13134A>G (Leu266Leu)
	m.13708G>A (Ala458Thr)
	m.13759G>A (Ala475Thr)
MT-CYB	m.14766C>T (Ile7Thr)
	m.15148G>A (Pro134Pro)
	m.15257G>A (Asp171Asn)
	m.15326A>G (Thr194Leu)
	m.15452C>A (Leu236Ile)
	m.15679A>G (Lys311Lys)
MT-TT	
MT-DLOOP	m.16069C>T
	m.16126T>C
	m.73A>G
	m.150C>T
	m.195T>C
	m.263A>G
	m.295C>T
	m.310insC
	m.489T>C
	m.514del(CA)

BROTHER

Mutación	120931/ARP
MT-RNR1	m.750A>G
	m.1438A>G
MT-RNR2	m.2706A>G
MT-ND1	m.4216T>C (Tyr304His)
MT-ND2	m.4769A>G (Met100Met)
	m.5484A>G (Ile339Val)(heteroplasmia)
	m.5493T>C (Phe342Leu) (heteroplasmia)
MT-CO1	m.6671T>C (His256His)
	m.7028C>T (Ala375Ala)
	m.7076A>G (Gly391Gly)
MT-CO2	
MT-TS1	m7476C>T
MT-ATP6	m.8860A>G (Thr112Ala)
	m.8958C>T (Ile144Ile)
MT-CO3	
MT-ND3	m.10398A>G (Thr114Ala)
MT-ND4L	m.10499A>G (Leu10Leu)
MT-ND4	m.11002A>G (Gln81Gln)
	m.11016G>A (Ser86Asn)
	m.11251A>G (Leu164Leu)
	m.11377G>A (Lys206Lys)
	m.11719G>A (Gly320Gly)
MT-TL2	
MT-ND5	m.12570A>G (Leu78Leu)
	m.12612A>G (Val92Val)
	m.13134A>G (Leu266Leu)
	m.13708G>A (Ala458Thr)
	m.13759G>A (Ala475Thr)
MT-CYB	m.14766C>T (Ile7Thr)
	m.15148G>A (Pro134Pro)
	m.15257G>A (Asp171Asn)
	m.15326A>G (Thr194Leu)
	m.15452C>A (Leu236Ile)
	m.15679A>G (Lys311Lys)
MT-TT	
MT-DLOOP	m.16069C>T
	m.16126T>C
	m.73A>G
	m.150C>T
	m.195T>C
	m.263A>G
	m.295C>T
	m.310insC
	m.489T>C
	m.514del(CA)

Supplementary figure 3: mtDNA sequence from Patient 2

Mutación	120936/ANA
MT-RNR1	m.750A>G
	m.1438A>G
MT-RNR2	m.2706A>G
	m.3010G>A
MT-ND1	m.3394T>C (Tyr30His)
	m.4216T>C (Tyr304His)
MT-ND2	m.4769A>G (Met100Met)
MT-CO1	m.7028>T (Ala375Ala)
	m.7142T>C (His413His)
	m.7184A>G (Pro427Pro)
MT-CO2	
MT-TS1	
MT-ATP6	m.8860A>G (Thr112Ala)
MT-CO3	m.9531A>G (Thr109Ala)
	m.9575G>A (Pro123Pro)
MT-ND3	m.10398A>G (Thr114Ala)
MT-ND4L	
MT-ND4	m.11251A>G (Leu164Leu)
	m.11719G>A (Gly320Gly)
MT-TL2	
MT-ND5	m.12612A>G (Val92Val)
	m.13708G>A (Ala458Thr)
MT-CYB	m.14766C>T (Ile7Thr)
	m.14798T>C (Phe18Leu)
	m.15326A>G (Thr194Leu)
	m.15452C>A (Leu236Phe)
MT-TT	
MT-DLOOP	m.16069C>T
	m.16126T>C
	m.16311T>C
	m.73A>G
	m.185G>A
	m.228G>A
	m.263A>G
	m.295C>T
	m.310insC
	m.462C>T
	m.482T>C
	m.489T>C

Figure S4. mtDNA sequence from family of patient 2.

FATHER MOTHER BROTH

Mutación	120938/Francisco
MT-RNR1	m.750A>G
	m.1438A>G
MT-RNR2	
MT-ND1	m.3777T>C (Ser157Ser)
	m.3915G>A (Gly203Gly)
MT-ND2	m.4727A>G (Met86Met)
	m.4769A>G (Met100Met)
MT-CO1	
MT-CO2	m.7805G>A (Val74Ile)
MT-TS1	
MT-ATP6	m.8860A>G (Thr112Ala)
MT-CO3	m.9380G>A (Trp58Trp)
MT-ND3	
MT-ND4L	
MT-ND4	m.11253T>C (Ile165Thr)
MT-TL2	
MT-ND5	
MT-CYB	m.15326A>G (Thr194Ala)
MT-TT	
MT-DLOOP	m.16362T>C
	m.16482A>G
	m.150C>T
	m.239T>C
	m.263A>G
	m.302insCC
	m.310insC
	m.514ins(CA)

Mutación	120937/Lorenza
MT-RNR1	m.750A>G
	m.1438A>G
MT-RNR2	m.2706A>G
	m.3010G>A
MT-ND1	m.3394T>C (Tyr30His)
	m.4216T>C (Tyr304His)
MT-ND2	m.4769A>G (Met100Met)
MT-CO1	m.7028C>T (Ala375Ala)
	m.7142T>C (His413His)
	m.7184A>G (Pro427Pro)
MT-CO2	
MT-TS1	
MT-ATP6	m.8860A>G (Thr112Ala)
MT-CO3	m.9531A>G (Thr109Ala)
	m.9575G>A (Pro123Pro)
MT-ND3	m.10398A>G (Thr114Ala)
MT-ND4L	
MT-ND4	m.11251A>G (Leu164Leu)
	m.11719G>A (Gly320Gly)
MT-TL2	
MT-ND5	m.12612A>G (Val92Val)
	m.13708G>A (Ala458Thr)
MT-CYB	m.14766C>T (Ile7Thr)
	m.14798T>C (Phe18Leu)
	m.15326A>G (Thr194Leu)
	m.15452C>A (Leu236Phe)
MT-TT	
MT-DLOOP	m.16069C>T
	m.16126T>C
	m.16311T>C
	m.73A>G
	m.185G>A
	m.228G>A
	m.263A>G
	m.295C>T
	m.310insC
	m.462C>T
	m.482T>C
	m.489T>C

Mutación	120939/Jose Antonio
MT-RNR1	m.750A>G
	m.1438A>G
MT-RNR2	m.2706A>G
	m.3010G>A
MT-ND1	m.3394T>C (Tyr30His)
	m.4216T>C (Tyr304His)
MT-ND2	m.4769A>G (Met100Met)
MT-CO1	m.7028C>T (Ala375Ala)
	m.7142T>C (His413His)
	m.7184A>G (Pro427Pro)
MT-CO2	
MT-TS1	
MT-ATP6	m.8860A>G (Thr112Ala)
MT-CO3	m.9531A>G (Thr109Ala)
	m.9575G>A (Pro123Pro)
MT-ND3	m.10398A>G (Thr114Ala)
MT-ND4L	
MT-ND4	m.11251A>G (Leu164Leu)
	m.11719G>A (Gly320Gly)
MT-TL2	
MT-ND5	m.12612A>G (Val92Val)
	m.13708G>A (Ala458Thr)
MT-CYB	m.14766C>T (Ile7Thr)
	m.14798T>C (Phe18Leu)
	m.15326A>G (Thr194Leu)
	m.15452C>A (Leu236Phe)
MT-TT	
MT-DLOOP	m.16069C>T
	m.16126T>C
	m.16311T>C
	m.73A>G
	m.185G>A
	m.228G>A
	m.263A>G
	m.295C>T
	m.310insC
	m.462C>T
	m.482T>C
	m.489T>C
